# An Intervention for Changing Sedentary Behavior Among African Americans With Multiple Sclerosis: Protocol

**DOI:** 10.2196/12973

**Published:** 2019-05-01

**Authors:** Jessica F Baird, Jeffer Eidi Sasaki, Brian M Sandroff, Gary Cutter, Robert W Motl

**Affiliations:** 1 University of Alabama at Birmingham Birmingham, AL United States; 2 Federal University of Triangulo Mineiro Uberaba Brazil

**Keywords:** African Americans, multiple sclerosis, sedentary behavior, intervention

## Abstract

**Background:**

Sedentary behavior is a major concern among patients with multiple sclerosis (MS), as it may accelerate disease progression and exacerbate physical disability. This is especially concerning among African Americans, a segment of the MS population who present with greater neurological disability and higher odds of physical comorbidities than their Caucasian counterparts.

**Objective:**

To date, researchers have not proposed interventions that focus on changing sedentary behavior in African Americans with MS.

**Methods:**

This paper describes a pilot study that examines the feasibility and efficacy of using text messaging along with theory-driven newsletters and behavioral coaching for changing sedentary behavior in African Americans with MS. We herein present the methods, procedures, and outcomes for our ongoing study.

**Results:**

Enrollment began in February 2018 and is expected to conclude in April 2019. Study results will be reported in the fall of 2019.

**Conclusions:**

After completion of this pilot intervention, we will summarize our study results in manuscripts for publication in peer-reviewed journals that will provide critical information on the feasibility and efficacy of our strategy. These results will inform future studies and, potentially, larger interventions for remotely reducing sedentary behavior in African Americans with MS.

**Trial Registration:**

ClinicalTrials.gov NCT03671499; https://clinicaltrials.gov/ct2/show/NCT03671499 (Archived by WebCite at http://www.webcitation.org/77MZnxyNy)

**International Registered Report Identifier (IRRID):**

DERR1-10.2196/12973

## Introduction

Sedentary behavior is defined as any waking activity performed in a seated or lying position with energy expenditure ≤1.5 metabolic equivalents of task (MET), with 1 MET being the metabolic rate at rest [1]. Sedentary behavior represents a major public health concern based on associations with morbidity and mortality [2,3], independent of physical activity [1].

Sedentary behavior has recently received attention among persons with the chronic, disabling disease, multiple sclerosis (MS) [4]. Sedentary behavior is two times higher in MS than in the general population, and sitting time increases across levels of worsening MS-related mobility disability [5]. Such an association likely involves sedentary behavior inducing comorbid conditions [6] that can accelerate disease progression [4] and ultimately worsen MS disability over time. This may be particularly pertinent for African Americans with MS. This group demonstrates worse neurological disability [7] and increased odds of physical comorbidities compared with Caucasian counterparts [8]. There is evidence that African Americans with MS experience a more aggressive disease course and present a blunted response to disease modifying therapies [9]. There are no data on racial differences in sedentary behavior in MS, but evidence in the general population indicates that African Americans spend more time sitting than Caucasians [10].

We recently reported that only 1.7% of participants in studies of exercise and physical activity in persons with MS were African American [11]. Furthermore, there is no research on the management of MS through reduction of sedentary behavior in African Americans with MS [12]. This may be associated with barriers toward implementing behavior change interventions in African Americans, such as diminished health care accessibility based on socioeconomic disparities and greater likelihood of living in low-income neighborhoods [13,14] where rehabilitation centers and facilities are often inaccessible. The use of electronic technology may be part of a solution for this problem and represents a strategy that can be applied for increasing the reach of behavior change interventions by allowing intervention delivery remotely, reducing participant burden, offering access, and reducing implementation costs and research personnel burden [15]. Accordingly, text messaging represents a promising medium for reaching a large number of persons remotely, as 94% of Americans own a mobile phone [16]. This strategy has already been used for promoting changes in health behaviors (eg, eating habits and medication use) and preventing chronic diseases (eg, cardiovascular disease and diabetes) in other populations [17-23]. We therefore believe that change in sedentary behavior might be facilitated by text messaging and newsletters, supplemented with one-on-one behavioral coaching. Studies have further reported that text messaging interventions have resulted in sedentary behavior reductions and increases in physical activity in the general population [17,18,24,25], yet we are unaware of any studies using text messaging to reduce sedentary behavior in African Americans with MS.

Text messaging is a promising strategy for reducing sedentary behavior in MS, and the literature indicates that anchoring intervention content with a behavior change theory likely increases the chance of success [26]. Our group has demonstrated that Social Cognitive Theory (SCT) has been effective in promoting changes in sedentary behavior and physical activity among persons with MS [27-29]. SCT provides a good framework for guiding changes in health behavior, as it includes well-tested principles and assumptions from different fields that investigate human behavior, such as psychology, anthropology, and sociology [30]. SCT further identified targets of behavior change, for example self-efficacy, outcome expectations, and goal setting, for designing the content of an intervention.

In view of the current evidence and literature gaps, we developed a technology-based behavior change intervention for reducing sedentary behavior in African Americans with MS. The intervention combines text messaging, print newsletters, and telephone-based coaching, all informed by SCT for reducing sedentary behavior in African Americans with MS. The proposed intervention will consist of a 12-week program divided into two phases: Phase 1 targets breaking up and reducing sedentary behavior and Phase 2 aims to reduce sedentary behavior by replacing it with light physical activity. To that end, sedentary behavior is the primary outcome of our intervention, but we will secondarily target increases in physical activity as a means of replacing sedentary behavior. The strategy of primarily focusing on a single behavior is based on the need of having a well-defined target for a successful intervention [26,31]. The project will examine the feasibility (ie, process, resources, management, and science) and preliminary efficacy (ie, changes in volume and patterns of sedentary behavior) of this intervention approach for interrupting and reducing sedentary behavior. The lessons learned from this study will inform the development of future large-scale interventions targeting reductions of sedentary behavior in African Americans with MS. The current protocol paper reports on the development, methodology, and outcome measures of this ongoing project.

## Methods

### Study Design and Overview

**Figure 1:**
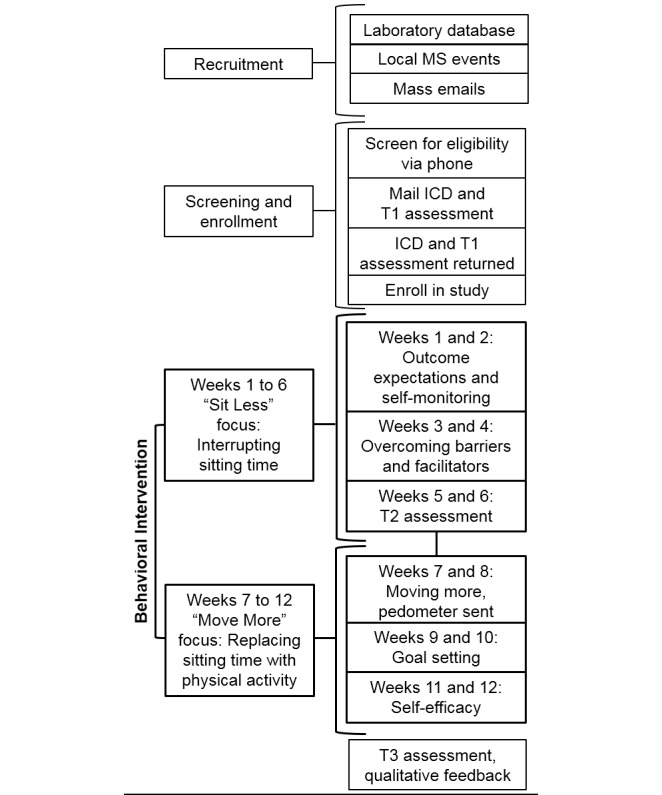
Flowchart outlining the program timeline from recruitment through completion of the program. MS: multiple sclerosis; ICD: informed consent document; T1: baseline; T2 midpoint; T3: postintervention.

The proposed study takes place at the University of Alabama at Birmingham (UAB), United States, and was designed based on guidelines for feasibility studies [32]. The study will use a single-group, pre-post intervention design that examines multiple domains of feasibility, including scientific efficacy of a 12-week, technology-based behavior change intervention for reducing sedentary behavior among African Americans with MS (ie, the *Sit Less, Move More* program). Feasibility will be assessed under four distinct domains: process, resources, management, and scientific metrics [33-35]. The primary scientific metric will be a change in sedentary behavior, measured both objectively with activity monitors and subjectively with self-report questionnaires. Secondary outcomes will include a change in physical activity and health-related quality of life. We will recruit a sample of 30 African American adults with MS. After being screened for eligibility, participants will be sent the informed consent document and the baseline assessment materials—questionnaires and activity monitors—via postal mail along with a preaddressed, prestamped envelope for returning the materials. After the baseline (T1) assessment, participants will start the 12-week intervention, which is divided into two phases: Phase 1 (weeks 1-6) targets breaking up and reducing sedentary behavior and Phase 2 (weeks 7-12) targets replacing sedentary behavior with physical activity. The intervention itself was developed through stakeholder involvement and consists of daily text messages and biweekly newsletters and telephone calls with a behavioral coach. The content of the text messages, newsletters, and coaching sessions will include SCT-based strategies for behavior change, as these approaches have successfully induced behavior change in home-based programs among persons with MS [30,36,37]. Midpoint (T2) and postintervention (T3) assessments will be conducted during week 6 and immediately after week 12, respectively. Participant flow from recruitment through completion of the program is depicted in Figure 1. Participants will be compensated US $25 for completing each of the three outcome assessment periods. Ethical approval to undertake the study has been obtained from the UAB Institutional Review Board, and the study has been registered with ClinicalTrials.gov (NCT03671499).

We will identify potential participants from those who have contacted our laboratory regarding research opportunities and who meet our inclusion criteria, outlined below, regarding race and age. An email will be distributed among those potential participants, followed by a phone call among those who do not respond to the initial email. We will also recruit individuals from the community at events sponsored by regional chapters of the National MS Society and other local organizations. If necessary, based on enrollment success, mass emails providing information about the study will be sent through the National MS Society, the North American Research Committee on Multiple Sclerosis registry, and iConquerMS. Those who are interested in participating will be instructed to call the laboratory, and authorized personnel will describe the study objectives and procedures. If a prospective participant continues to express interest in the study, we will conduct a telephone screening to ensure that the prospective participant satisfies the inclusion and exclusion criteria. Based on previous feasibility studies in persons with MS, we aim to recruit 30 individuals to participate in this study [38,39]. As this study is designed to assess the feasibility of conducting the Sit Less, Move More program, rather than confirming the efficacy of the intervention, an a priori power analysis for estimating sample size was not performed.

Inclusion criteria for the proposed study are as follows: (1) African American; (2) participant-reported MS diagnosis; (3) relapse-free in the last 30 days; (4) ambulatory with or without assistance based on a Patient-Determined Disease Steps (PDDS) score of 0-5; (5) self-report daily engagement in sedentary behavior of ≥480 minutes per day; (6) health contribution score of <14 calculated from the Godin Leisure-Time Exercise Questionnaire [40]; (7) absence of major musculoskeletal problems and/or cardiovascular, cardiopulmonary, and/or metabolic diseases that are contraindications for changing physical activity and sedentary behavior levels; (8) living in the United States; and (9) ownership of a mobile phone capable of receiving text messages.

### Feasibility Metrics

Feasibility will be assessed based on four domains: process (eg, recruitment and retention rates), resources (eg, communication and monetary requirements), management (eg, researcher preparation and capacity), and science (eg, safety and efficacy of the intervention). To assess process-related feasibility, we will document recruitment, retention, and adherence rates. Recruitment will be evaluated as the number of individuals who initially expressed interest in participating and agreed to be screened for eligibility after receiving more information about the study. Retention will be evaluated as the number of participants retained from enrollment through completion of the study. We will maintain a log of the number of participants who complete the biweekly behavioral coaching sessions to determine adherence. To measure resource-related feasibility, we will maintain logs of time spent communicating with participants and of monetary costs involved in the conduction of the study. Management-related feasibility will be evaluated by maintaining a log indicating the efforts and time spent by research personnel related to collecting and entering data, maintaining equipment and research-related material, and managing the study logistics. Safety and efficacy of the intervention will be included as scientific feasibility metrics. To assess safety, we will maintain records of any adverse or serious adverse events and any medical concerns reported by the participants. We will follow standard UAB protocols in the reporting of any such events. The primary scientific outcome is a change in sedentary behavior. Secondary scientific outcomes include changes in physical activity and quality of life (see Behavioral Outcomes section for more detail). After completing the study, the participants will provide feedback regarding satisfaction and personal experiences with the study using a survey with both Likert scale questions and open-ended questions. This information, along with the results of the feasibility analysis, will be valuable for developing larger-scale studies designed to establish the efficacy and effectiveness of this treatment approach and to identify future strategies of effective behavior change.

### Behavioral Outcomes

Participants will provide information on clinical and demographic characteristics (eg, disease course and duration, education level, and level of income) and disability status (ie, PDDS) during the baseline (T1) assessment period. The primary behavioral outcome will include a change in sedentary behavior, with changes in physical activity and health-related quality of life assessed as secondary outcomes. All outcomes will be measured at three different time points: baseline (T1 assessment), midpoint (T2 assessment), and postintervention (T3 assessment). Sedentary behavior and physical activity behavior will be measured objectively with accelerometry, using the activPAL activity monitor (PAL Technologies) and the ActiGraph model GT3X+ activity monitor (ActiGraph LLC). During each assessment time point, participants will wear both activity monitors for a 7-day period, only removing them for sleeping or during water-based activities (eg, bathing and swimming). The activPAL monitor will be worn at the middle of the anterior aspect of the thigh and the ActiGraph will be worn on an elastic belt around the waist at the nondominant hip. Standardized instructions describing how to wear the activity monitors, including picture references, will be provided at each assessment time point. Data obtained from the activPAL will be classified as time spent sitting or lying down, standing, or during movement using a proprietary algorithm; these metrics represent the primary sedentary behavior outcome [41]. The activPAL data will further indicate how frequently sitting is interrupted through the number of sit-to-stand transitions. Sedentary behavior measures from the ActiGraph device will supplement measures from the activPAL. The ActiGraph data will be assessed as 60-second epochs and physical activity behavior will be defined by established cut points for persons with MS: sedentary behavior is <100 activity counts per minute, light physical activity is between 100 and 1583 activity counts per minute, and moderate-to-vigorous physical activity is ≥1584 activity counts per minute [42]. The number of minutes spent at each activity level (ie, sedentary vs light physical activity vs moderate-to-vigorous physical activity) will be the primary outcome from the ActiGraph data. In addition, sedentary behavior interruption rate will be assessed from ActiGraph data as the number of transitions from <100 activity counts per minute to ≥100 activity counts per minute. In addition to the objective accelerometry measures, subjective self-report measures of sedentary behavior and physical activity will be completed using the Godin Leisure-Time Exercise Questionnaire [40,43] and the International Physical Activity Questionnaire short version [44,45]. The 36-item Short Form Health Survey (SF-36) [46] will be completed at each measurement time point for self-reported measurement of physical and mental indices of health-related quality of life.

### Behavioral Intervention

The intervention will be 12 weeks in duration and divided into two, 6-week phases. Phase 1 (weeks 1-6) will focus on interrupting long periods of sedentary behavior, and Phase 2 (weeks 7-12) will focus on replacing sedentary behavior with light physical activity. Participants will be mailed biweekly (ie, all odd-numbered weeks) SCT-based newsletters that highlight ways of overcoming difficulties in effectively interrupting sedentary behavior and replacing sedentary behavior with physical activity; see Figure 1 for a week-by-week list of SCT-based themes. For example, newsletters focus on anticipating outcomes and setting goals, which are characteristic features of human agency, one of the primary tenets of SCT [30]. We further highlight self-efficacy within the newsletters, as it is a focal determinant of health behavior change within SCT [47,48]. Within 5 days of receiving the newsletter, a trained behavioral coach will call the participant via telephone to discuss the information provided in the newsletter and review strategies for behavior change. Throughout the 12-week intervention period, participants will receive two daily text messages, Monday through Friday, which will be sent in the morning and the afternoon and contain content relevant to the topic being reviewed that week. For example, a week 1 (*Outcome Expectations and Self-Monitoring*) text message will read, “Expectations are important for sticking with your plan to reduce sitting time. Remember to look up the benefits of sitting less,” and a week 7 (*Moving More*) text message will read, “Try to walk around the house during TV commercials.” Text messages will be standardized across participants. At the outset of the intervention, participants will be provided with a journal to log behavior and monitor progress. This journal is provided as a self-monitoring tool rather than a method of strictly tracking all sedentary behavior and physical activity. The purpose of the journal is to help the participant (1) recognize how much time is spent sitting, (2) identify opportunities to reduce sitting, and (3) track progress throughout the program. During the first phase of the study (weeks 1-6), participants will be encouraged to record time spent sitting. Immediately following the first phase of the study (ie, beginning of week 7), participants will be provided with a Digi-Walker SW200 pedometer (Yamax) for tracking daily step counts. This is an important study aspect, given that the second phase of the study involves the program transitioning from *sitting less* to *moving more*. To that end, during the second phase of the study (weeks 7-12), participants will be encouraged to record time spent physically active, including the number of steps per day, in the provided journal.

Of note, while developing the study materials, we engaged in the process of stakeholder feedback through an informal focus group with five African American members of a local MS support group. We presented the study materials (ie, text messages, newsletters, and journals) to the focus group members and asked them to provide comments and suggestions for improving the material content. We obtained direct feedback on the content and vocabulary of the text messages, newsletters, and journals. Participants reached a consensus that the materials needed lay vocabulary. In terms of the text messages, each message was inspected for sensitive content and suitability to daily routine. The focus group suggested modifications to wording of some messages and to the feasibility of some activities that were deemed impractical (eg, “Eat while standing at the restaurant”). The suggestions were incorporated into the development of the patient-informed and culturally tailored final study materials.

### Data Analysis

Feasibility data for process, resources, and management will be examined via percentage, frequency analysis, and descriptive statistics. Regarding scientific outcomes, data normality will be verified using the Shapiro-Wilk test and descriptive statistics will be computed for all variables per each assessment point. The influence of the intervention on sedentary behavior, physical activity, and physical function—a component of the SF-36—will be evaluated using repeated-measures linear mixed models, respecting the interdependence of measures over time. Point-by-point improvements in quality of life related to reductions in sedentary behavior and increases in physical activity will be assessed using estimates at each measurement time point, adjusted for sociodemographic and clinical covariates. We will further conduct other exploratory analyses as permitted by the data.

## Results

Enrollment began in February 2018 and is expected to conclude in April 2019. Intervention delivery will conclude in August 2019. Data analysis with full study results is expected in the fall of 2019.

## Discussion

This study will identify the demands and procedures of the proposed intervention strategy (ie, text messaging, newsletters, and behavioral coaching) for changing sedentary behavior in African Americans with MS. The knowledge acquired from this intervention will be valuable in designing future studies for reducing sedentary behavior in a larger number of African Americans with MS. We expect that our intervention will promote reductions and changes in the volume and pattern of sedentary behavior and, by extension, an increase in light physical activity in the study participants. It is important to highlight that, even though the intervention focuses on both sedentary behavior and physical activity, we purposefully place the emphasis on the former, as the literature indicates that intervention success requires a well-defined target [26,31].

The target criterion for considering the intervention a success will be a reduction in sitting time of 60+ minutes per day, as there is evidence that replacement of one hour per day of sitting with physical activity of any intensity can improve quality of life [49]. Studies have further adopted this same volume of sedentary behavior reduction, indicating that it is a reasonable goal for an intervention [24,50]. If the data analyses indicate that this intervention is both feasible (ie, adequate results for process, resources, management, and science) and efficacious (ie, sedentary behavior reductions of 60+ minutes per day), the study results may provide practical and scientific support for applying such an approach in subsequent, large-scale studies that can efficiently target a large number of African Americans with MS who engage in high levels of sedentary behavior (ie, go/no-go decision for a subsequent trial).

The use of technology for reducing sedentary behavior represents an important step for reaching persons with MS remotely [27] and for promoting behavior changes that may improve health [21,51,52]. As of 2018, almost every American (ie, 94%) owned a mobile phone [16], and this makes text messaging an optimal medium for disseminating health information, including information that is directed toward educating people about changing behaviors. The examination of whether text messaging, coupled with the provision of SCT-based newsletters and behavior coaching, may help change sedentary behavior in African Americans with MS is central for scaling up the intervention among a larger number of persons from this population group who might not have access to resources for engaging in regular interventions and/or in-person behavioral intervention. Furthermore, such an approach is advantageous for optimizing the intervention based on the socioeconomic and cultural reality of African Americans with MS. For example, all the intervention materials were designed based on specific characteristics of the target population, as per the recommendations and feedback from a focus group of African American persons with MS (ie, stakeholder engagement). This process contributed toward creating cultural awareness among the researchers and developing strategies appropriate for the target population to maximize the potential to reduce sedentary behavior with the intervention.

There is evidence of the increasing use of technology in theory-based behavior change interventions in people with disabilities, including MS [15]. These strategies have demonstrated promising results in increasing exercise and physical activity levels in such groups of people [15]. The use of technology is ideal for promoting behavior change remotely in large groups of people, given its affordability, reduced personnel burden, and lack of reliance on physical space [20,51,52]. The use of technology can further minimize or even eliminate in-person attendance at specific physical spaces (eg, gyms and recreational clubs) [27-29]. By reducing the burden on both researchers and participants, remote interventions enhance the feasibility and likelihood of successful uptake and adoption of interventions that reduce sedentary behavior. To date, researchers have used computer software [53], DVD-delivered interventions [27,28], and text messaging [24] for promoting reductions in sedentary behavior in the general population and persons with MS. Our study will be a further step in amplifying the possibilities of promoting changes in sedentary behavior remotely in large groups of underserved persons with MS, such as African Americans.

Another advantageous tenet of our intervention involves the particularly high frequency of stimuli for promoting the intended changes in behavior, as text messages can be sent more than once per day over different time periods. This is critical in reminding individuals about continually monitoring sitting behavior and identifying ways to interrupt it. Sedentary behavior is a highly prevalent behavior during a regular day that is difficult to self-monitor. It is usually easier to remember activities that are more intense and less frequent during the day (eg, sports, dance, and recreational activities) [54]. As such, increased frequency of stimuli may be important for effectively reducing sedentary behavior. The results may provide further directions to more efficaciously target this health behavior in future studies.

One strength of this study is the use of both the activPAL and ActiGraph monitors to assess sedentary behavior and physical activity. We will be able to cross-examine the estimates for agreement as well as provide sedentary behavior estimates based on both posture and lack of movement. The inclusion of both devices might further provide data on acceptability and preferences for a device for future trials. Some limitations of this study are noteworthy. The study sample will not be representative of all African Americans with MS since we will only recruit a small number of participants (ie, 30 participants). Nevertheless, the results will provide an indication of whether the proposed strategy has the potential to be delivered and efficacious in a larger sample of African Americans with MS. Another limitation is the short intervention duration; the entire intervention will last for only 3 months. Yet, behavior change is usually considered stable when maintained for longer than 6 months. Funding limitations did not permit collection of such data, but we may be able to obtain data regarding behavior change maintenance (ie, 6+ months) if funding opportunities arise. The study will not include a control condition. This would be important for ensuring that any behavior changes are due to our intervention itself and not due to other factors; this will be important for future, larger-scale interventions. Another possibility would be to include a comparison group comprised of Caucasians with MS, as this would provide insight regarding differences in responses between African Americans and Caucasians with MS. One final limitation of the study is the lack of a double baseline assessment week. This would be important for assessing reactivity to wearing the activity monitors and, therefore, to better isolate the effects of the intervention on the primary study outcomes. Lastly, we did not examine, in depth, the level of familiarization of participants with portable electronic technology. If more complex electronic strategies are used for prompting behavior change, then researchers need to consider participants’ prior history of mobile technology usage for expanding the use of such technology in changing behavior in MS.

In summary, this paper describes the methods and outcomes of interest of a technology-based intervention aimed at reducing sedentary behavior in African Americans with MS. This pilot study will provide data on the feasibility and efficacy of our strategy in promoting sedentary behavior reductions in the aforementioned group and we anticipate the results will be important for researchers investigating strategies for reducing sedentary behavior in MS. After completion of this pilot intervention, we will summarize our study results in manuscripts for publication in peer-reviewed journals that will provide critical information on the feasibility and efficacy of our strategy. These results will inform future studies and, potentially, larger interventions for remotely reducing sedentary behavior in African Americans with MS.

## Abbreviations

ICD: informed consent document
MET: metabolic equivalents of task
MS: multiple sclerosis
PDDS: Patient-Determined Disease Steps
SCT: Social Cognitive Theory
SF-36: 36-item Short Form Health Survey
T1: baseline
T2: midpoint
T3: postintervention
UAB: University of Alabama at Birmingham

## References

[1] Owen N, Healy GN, Matthews CE, Dunstan DW (2010). Too much sitting: The population health science of sedentary behavior. Exerc Sport Sci Rev.

[2] de Rezende LF, Rodrigues Lopes M, Rey-López JP, Matsudo VK, do Carmo Luiz O (2014). Sedentary behavior and health outcomes: An overview of systematic reviews. PLoS One.

[3] Young DR, Hivert MF, Alhassan S, Camhi SM, Ferguson JF, Katzmarzyk PT, Lewis CE, Owen N, Perry CK, Siddique J, Yong CM, Physical Activity Committee of the Council on Lifestyle and Cardiometabolic Health; Council on Clinical Cardiology; Council on Epidemiology and Prevention; Council on Functional Genomics and Translational Biology; and Stroke Council (2016). Sedentary behavior and cardiovascular morbidity and mortality: A science advisory from the American Heart Association. Circulation.

[4] Veldhuijzen van Zanten JJ, Pilutti LA, Duda JL, Motl RW (2016). Sedentary behaviour in people with multiple sclerosis: Is it time to stand up against MS?. Mult Scler.

[5] Sasaki JE, Motl RW, Cutter G, Marrie RA, Tyry T, Salter A (2018). National estimates of self-reported sitting time in adults with multiple sclerosis. Mult Scler J Exp Transl Clin.

[6] Hubbard EA, Motl RW, Fernhall B (2018). Sedentary behavior and blood pressure in patients with multiple sclerosis. Int J MS Care.

[7] Kaufman MD, Johnson SK, Moyer D, Bivens J, Norton HJ (2003). Multiple sclerosis: Severity and progression rate in African Americans compared with whites. Am J Phys Med Rehabil.

[8] Marrie RA, Horwitz R, Cutter G, Tyry T, Campagnolo D, Vollmer T (2008). Comorbidity, socioeconomic status, and multiple sclerosis. Mult Scler.

[9] Klineova S, Nicholas J, Walker A (2012). Response to disease modifying therapies in African Americans with multiple sclerosis. Ethn Dis.

[10] Thomas BE, Charles N, Watson B, Chandrasekaran V, Senthil Kumar R, Dhanalakshmi A, Wares F, Swaminathan S (2015). Prevalence of chest symptoms amongst brick kiln migrant workers and care seeking behaviour: A study from South India. J Public Health (Oxf).

[11] Lai B, Cederberg K, Vanderbom KA, Bickel CS, Rimmer JH, Motl RW (2018). Characteristics of adults with neurologic disability recruited for exercise trials: A secondary analysis. Adapt Phys Activ Q.

[12] Khan O, Williams MJ, Amezcua L, Javed A, Larsen KE, Smrtka JM (2015). Multiple sclerosis in US minority populations: Clinical practice insights. Neurol Clin Pract.

[13] Kershaw KN, Albrecht SS, Carnethon MR (2013). Racial and ethnic residential segregation, the neighborhood socioeconomic environment, and obesity among Blacks and Mexican Americans. Am J Epidemiol.

[14] Lewis CE, Raczynski JM, Heath GW, Levinson R, Hilyer JJ, Cutter GR (1993). Promoting physical activity in low-income African-American communities: The PARR project. Ethn Dis.

[15] Lai B, Young HJ, Bickel CS, Motl RW, Rimmer JH (2017). Current trends in exercise intervention research, technology, and behavioral change strategies for people with disabilities: A scoping review. Am J Phys Med Rehabil.

[16] (2018). Pew Research Center.

[17] Armanasco AA, Miller YD, Fjeldsoe BS, Marshall AL (2017). Preventive health behavior change text message interventions: A meta-analysis. Am J Prev Med.

[18] Cole-Lewis H, Kershaw T (2010). Text messaging as a tool for behavior change in disease prevention and management. Epidemiol Rev.

[19] Park LG, Beatty A, Stafford Z, Whooley MA (2016). Mobile phone interventions for the secondary prevention of cardiovascular disease. Prog Cardiovasc Dis.

[20] Palmer MJ, Barnard S, Perel P, Free C (2018). Mobile phone-based interventions for improving adherence to medication prescribed for the primary prevention of cardiovascular disease in adults. Cochrane Database Syst Rev.

[21] Anglada-Martinez H, Riu-Viladoms G, Martin-Conde M, Rovira-Illamola M, Sotoca-Momblona J, Codina-Jane C (2015). Does mHealth increase adherence to medication? Results of a systematic review. Int J Clin Pract.

[22] Ramirez M, Wu S, Beale E (2016). Designing a text messaging intervention to improve physical activity behavior among low-income Latino patients with diabetes: A discrete-choice experiment, Los Angeles, 2014-2015. Prev Chronic Dis.

[23] Joiner KL, Nam S, Whittemore R (2017). Lifestyle interventions based on the diabetes prevention program delivered via eHealth: A systematic review and meta-analysis. Prev Med.

[24] Cotten E, Prapavessis H (2016). Increasing nonsedentary behaviors in university students using text messages: Randomized controlled trial. JMIR Mhealth Uhealth.

[25] Downing KL, Salmon J, Hinkley T, Hnatiuk JA, Hesketh KD (2017). A mobile technology intervention to reduce sedentary behaviour in 2- to 4-year-old children (Mini Movers): Study protocol for a randomised controlled trial. Trials.

[26] Baranowski T, Cullen KW, Nicklas T, Thompson D, Baranowski J (2003). Are current health behavioral change models helpful in guiding prevention of weight gain efforts?. Obes Res.

[27] Klaren RE, Hubbard EA, Motl RW (2014). Efficacy of a behavioral intervention for reducing sedentary behavior in persons with multiple sclerosis: A pilot examination. Am J Prev Med.

[28] McAuley E, Wójcicki TR, Learmonth YC, Roberts SA, Hubbard EA, Kinnett-Hopkins D, Fanning J, Motl RW (2015). Effects of a DVD-delivered exercise intervention on physical function in older adults with multiple sclerosis: A pilot randomized controlled trial. Mult Scler J Exp Transl Clin.

[29] Adamson BC, Learmonth YC, Kinnett-Hopkins D, Bohri M, Motl RW (2016). Feasibility study design and methods for Project GEMS: Guidelines for Exercise in Multiple Sclerosis. Contemp Clin Trials.

[30] Motl RW, Pekmezi D, Wingo BC (2018). Promotion of physical activity and exercise in multiple sclerosis: Importance of behavioral science and theory. Mult Scler J Exp Transl Clin.

[31] Fishbein M, Yzer MC (2003). Using theory to design effective health behavior interventions. Commun Theory.

[32] Learmonth YC, Motl RW (2018). Important considerations for feasibility studies in physical activity research involving persons with multiple sclerosis: A scoping systematic review and case study. Pilot Feasibility Stud.

[33] Thabane L, Ma J, Chu R, Cheng J, Ismaila A, Rios LP, Robson R, Thabane M, Giangregorio L, Goldsmith CH (2010). A tutorial on pilot studies: The what, why and how. BMC Med Res Methodol.

[34] Lancaster GA (2015). Pilot and feasibility studies come of age!. Pilot Feasibility Stud.

[35] Lancaster GA, Dodd S, Williamson PR (2004). Design and analysis of pilot studies: Recommendations for good practice. J Eval Clin Pract.

[36] Suh Y, Weikert M, Dlugonski D, Sandroff B, Motl RW (2011). Social cognitive correlates of physical activity: Findings from a cross-sectional study of adults with relapsing-remitting multiple sclerosis. J Phys Act Health.

[37] Ellis T, Motl RW (2013). Physical activity behavior change in persons with neurologic disorders: Overview and examples from Parkinson disease and multiple sclerosis. J Neurol Phys Ther.

[38] Learmonth YC, Adamson BC, Kinnett-Hopkins D, Bohri M, Motl RW (2017). Results of a feasibility randomised controlled study of the guidelines for exercise in multiple sclerosis project. Contemp Clin Trials.

[39] Sebastião E, McAuley E, Shigematsu R, Adamson BC, Bollaert RE, Motl RW (2018). Home-based, square-stepping exercise program among older adults with multiple sclerosis: Results of a feasibility randomized controlled study. Contemp Clin Trials.

[40] Godin G, Shephard RJ (1985). A simple method to assess exercise behavior in the community. Can J Appl Sport Sci.

[41] Coote S, O'Dwyer C (2012). Comparative validity of accelerometer-based measures of physical activity for people with multiple sclerosis. Arch Phys Med Rehabil.

[42] Sandroff BM, Motl RW, Suh Y (2012). Accelerometer output and its association with energy expenditure in persons with multiple sclerosis. J Rehabil Res Dev.

[43] Sikes EM, Richardson EV, Cederberg KJ, Sasaki JE, Sandroff BM, Motl RW (2018). Use of the Godin leisure-time exercise questionnaire in multiple sclerosis research: A comprehensive narrative review. Disabil Rehabil.

[44] Craig CL, Marshall AL, Sjöström M, Bauman AE, Booth ML, Ainsworth BE, Pratt M, Ekelund ULF, Yngve A, Sallis JF, Oja P (2003). International physical activity questionnaire: 12-country reliability and validity. Med Sci Sports Exerc.

[45] Lee PH, Macfarlane DJ, Lam TH, Stewart SM (2011). Validity of the International Physical Activity Questionnaire Short Form (IPAQ-SF): A systematic review. Int J Behav Nutr Phys Act.

[46] Ware JE, Sherbourne CD (1992). The MOS 36-item short-form health survey (SF-36). I. Conceptual framework and item selection. Med Care.

[47] Bandura A (2001). Social cognitive theory: An agentic perspective. Annu Rev Psychol.

[48] Bandura A (2004). Health promotion by social cognitive means. Health Educ Behav.

[49] Balboa-Castillo T, León-Muñoz LM, Graciani A, Rodríguez-Artalejo F, Guallar-Castillón P (2011). Longitudinal association of physical activity and sedentary behavior during leisure time with health-related quality of life in community-dwelling older adults. Health Qual Life Outcomes.

[50] Lewis LK, Rowlands AV, Gardiner PA, Standage M, English C, Olds T (2016). Small Steps: Preliminary effectiveness and feasibility of an incremental goal-setting intervention to reduce sitting time in older adults. Maturitas.

[51] Bond DS, Thomas JG, Raynor HA, Moon J, Sieling J, Trautvetter J, Leblond T, Wing RR (2014). B-MOBILE--A smartphone-based intervention to reduce sedentary time in overweight/obese individuals: A within-subjects experimental trial. PLoS One.

[52] McCarroll R, Eyles H, Ni Mhurchu C (2017). Effectiveness of mobile health (mHealth) interventions for promoting healthy eating in adults: A systematic review. Prev Med.

[53] Evans RE, Fawole HO, Sheriff SA, Dall PM, Grant PM, Ryan CG (2012). Point-of-choice prompts to reduce sitting time at work: A randomized trial. Am J Prev Med.

[54] Baranowski T (1988). Validity and reliability of self-report measures of physical activity: An information-processing perspective. Res Q Exerc Sport.

